# Cellulose biosynthesis inhibitor isoxaben causes nutrient-dependent and tissue-specific Arabidopsis phenotypes

**DOI:** 10.1093/plphys/kiad538

**Published:** 2023-10-12

**Authors:** Michael Ogden, Sarah J Whitcomb, Ghazanfar Abbas Khan, Ute Roessner, Rainer Hoefgen, Staffan Persson

**Affiliations:** Copenhagen Plant Science Center, Department of Plant & Environmental Sciences, University of Copenhagen, Frederiksberg C 1871, Denmark; Max Planck Institute of Molecular Plant Physiology, Potsdam-Golm 14476, Germany; Max Planck Institute of Molecular Plant Physiology, Potsdam-Golm 14476, Germany; Cereal Crops Research Unit, USDA-ARS, Madison, WI 53726, USA; Department of Animal, Plant and Soil Sciences, School of Agriculture, Biomedicine and Environment, La Trobe University, Bundoora, VIC 3086, Australia; Research School of Biology, The Australian National University, Acton, Australian Capital Territory 2600, Australia; Max Planck Institute of Molecular Plant Physiology, Potsdam-Golm 14476, Germany; Copenhagen Plant Science Center, Department of Plant & Environmental Sciences, University of Copenhagen, Frederiksberg C 1871, Denmark; Joint International Research Laboratory of Metabolic & Developmental Sciences, State Key Laboratory of Hybrid Rice, SJTU-University of Adelaide Joint Centre for Agriculture and Health, School of Life Sciences and Biotechnology, Shanghai Jiao Tong University, Shanghai 200240, China

Dear Editor,

The plant cell wall is a sugar-rich matrix that surrounds every plant cell, providing structural support and acting as a first line of defense against pests and pathogens. As cell walls comprise the bulk of plant biomass, they are a major carbon sink and the source of many essential commodities, such as food, shelter, fuel, and clothing. Therefore, it is no surprise that cell walls are a major research topic, as knowledge gained can be harnessed to engineer plants with improved or tailored cell walls.

Cellulose is the major cell wall polysaccharide, made of long chains of glucose that act as the main load-bearing cell wall component. Cellulose is synthesized by plasma membrane–localized cellulose synthase (CESA) complexes, comprised of 18 CESA subunits and various accessory proteins (Pedersen et al. 2023). Cellulose biosynthesis inhibitors (CBIs) have been used for decades as important agricultural herbicides and also as invaluable molecular tools for studying cellulose regulation, as it is widely hypothesized that CBIs specifically target CESAs to inhibit cellulose synthesis (Tateno et al. 2016). Furthermore, CBIs are commonly used to induce cell wall weakening to characterize cell wall integrity sensing and damage response pathways (Denness et al. 2011; Engelsdorf et al. 2018). Despite being a focal tool for cell wall research, reports exist in the literature to suggest that CBIs may not function the way that we think they do.

The long-held belief that CBIs target CESAs stems from forward genetic screens, where seedlings that resisted CBIs were consistently identified as being CESA missense mutants (Scheible et al. 2001; Desprez et al. 2002; Harris et al. 2012; Shim et al. 2018; Huang et al. 2020). The mechanism of resistance has been interpreted as abolished CBI–CESA binding. It is difficult to directly test CBI–protein binding, as CESAs are notoriously recalcitrant to heterologous expression and purification due to their complex tertiary structure and assembly in a large plasma membrane–localized complex. An attempt to confirm CBI–CESA6 interaction was carried out with the CBI endosidin20 and analog endosidin20-1, revealing potential interaction in vitro using a drug affinity responsive target stability assay and microscale thermophoresis (Huang et al. 2020, 2021). Stringent approaches are still needed to definitively demonstrate CBI–CESA interaction.

Curiously, the missense mutations that confer CBI resistance occur seemingly at random across primary cell wall CESAs, with no consistent pattern in the modification of particular amino acids or posttranslational modification sites (Larson and McFarlane 2021). It is peculiar that although CBIs are structurally diverse compounds, they are thought to specifically target highly homologous CESAs, and *cesa* missense mutants rarely display cross-resistance to multiple CBIs (Larson and McFarlane 2021). To make matters more puzzling, monocots tend to exhibit significantly stronger tolerance to certain CBIs compared with dicots, including isoxaben (Heim et al. 1993; Brabham et al. 2018), C17 (Hu et al. 2019), and endosidin20 (Huang and Zhang 2021), despite having no obvious resistance mechanisms. There are multiple reports of non-CESA mutants that exhibit CBI tolerance, as 7 mutants for nuclear-encoded mitochondrion-localized proteins tolerate various CBIs via an unknown mechanism (Scheible et al. 2003; Nakagawa and Sakurai 2006; Vellosillo et al. 2013; Hu et al. 2016). Furthermore, isoxaben does not affect CESAs involved in secondary cell wall synthesis, despite their high homology to primary cell wall CESAs that are putatively targeted (Watanabe et al. 2018). These findings raise the important question of whether we truly understand how CBIs act to inhibit cellulose synthesis, especially in the model species Arabidopsis (*Arabidopsis thaliana*). As CBIs are prevalent molecular tools used to drive fundamental research relating to cell wall regulation, cell wall damage response, and mechanobiology, it is crucially important to fully understand CBI mechanisms of action.

While conducting growth assays with Arabidopsis (Col-0) on media containing varying nutrient concentrations in combination with isoxaben, we unexpectedly uncovered further evidence that isoxaben, and likely other CBIs, works in an unexpected manner.

Cellulose-deficient mutants develop a characteristic root phenotype when grown on media containing 60 mM nitrate, exhibited as increased stunting, swelling, and root hair induction (Hermans et al. 2010), referred to here as the root stunting and swelling response (RSSR). This phenotype is not induced when seedlings are grown on a lower concentration of 6 mM nitrate. The mechanism that drives the 60 mM nitrate-induced RSSR remains unknown. To study this phenomenon and gauge whether nitrate availability could directly modulate cellulose synthesis, we grew Arabidopsis on 1× MS media (M407, PhytoTech Labs) lacking nitrogen, potassium, and phosphorus and modified it to contain a final concentration of 1.35 mM phosphate (as KH_2_PO_4_) and either 6 mM KNO_3_ or 60 mM KNO_3_. A relatively low concentration of isoxaben (1.5 nM) was added to media in an effort to induce mild cellulose inhibition. Seedlings were grown under long-day growth conditions (16 h:8 h, 21°C:19°C, light:dark) with 100 *µ*mol/m^2^/s LED lighting. We were able to recapture the nitrate-dependent phenotype, as a strong RSSR was induced when isoxaben was added to 60 mM KNO_3_ media, whereas the RSSR was highly suppressed by 6 mM KNO_3_ media (Fig. 1A). The same results were observed when using NaNO_3_ as the nitrate source. To control for the potential influence of 60 mM K^+^ or Na^+^ in driving the RSSR, modified 1× MS media was prepared with varying concentrations of KCl or NaCl with isoxaben or mock, without added nitrate. Unlike the drastic RSSR observed when isoxaben was combined with 60 mM KNO_3_ or NaNO_3_, 60 mM KCl or NaCl did not induce an RSSR, causing only a slightly decreased primary root length when compared with growth on 6 mM KCl or NaCl with isoxaben (Fig. 1, A and B). When 60 mM KCl or 60 mM NaCl was combined with a relatively low concentration of NO_3_^−^ (6 mM KNO_3_) and isoxaben, the RSSR was induced. These results suggest that the isoxaben-induced RSSR is not due to ionic stress but rather is modulated in a nutrient-specific manner that is not solely driven by 60 mM NO_3_^−^. By simply modifying media nutrient composition, the RSSR can vary drastically despite maintaining a consistent concentration of isoxaben, as was also observed when comparing isoxaben dose response phenotypes between standard ½ MS and 1× MS media (Fig. 1C).

**Figure 1.**
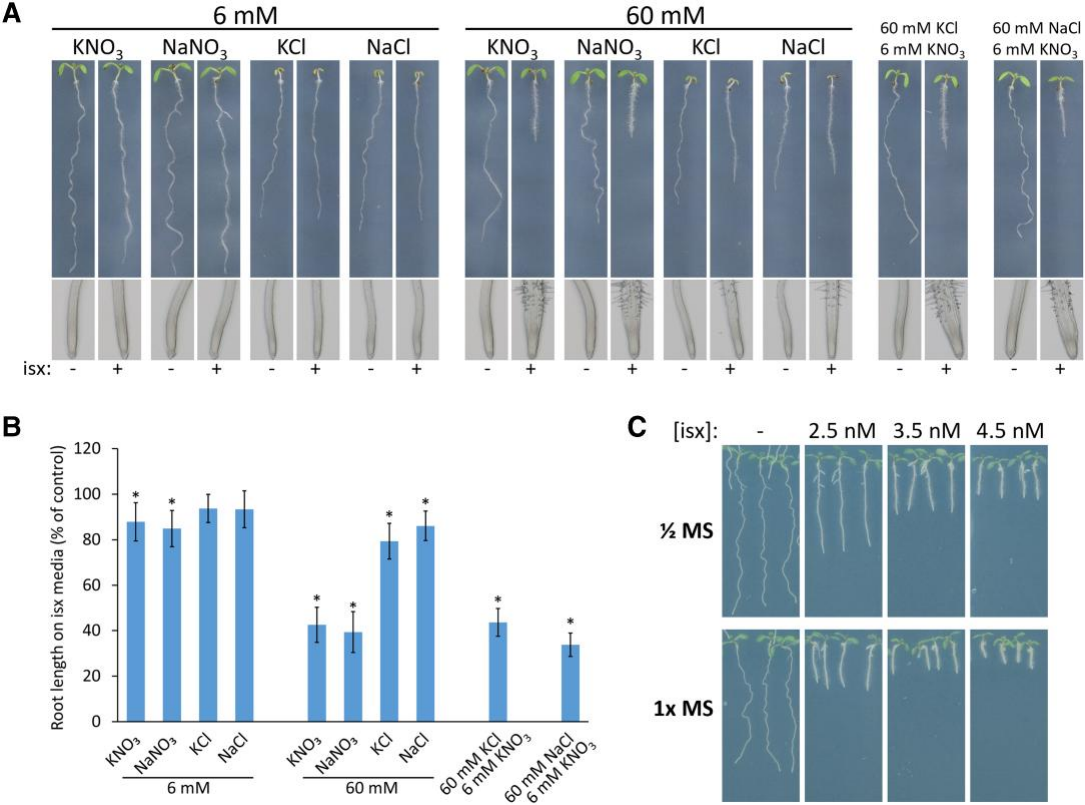
The phenotypic response to isoxaben is nutrient-dependent. **A)** Representative Col-0 root phenotypes 7 d after stratification. Seedlings were grown on 1× MS media (M407, PhytoTech Labs) lacking nitrogen, potassium, and phosphorus, modified to contain a final concentration of 1.35 mM phosphate (as KH_2_PO_4_) and each indicated nutrient, with 1.5 nM isoxaben (isx, +) or mock (ethanol, −). **B)** Isoxaben-induced change in root length, quantified from the growth assay in **A)**. Error bars represent means ± Sd (*n* = 9 to 29). Asterisk denotes significant difference from control (*P* < 0.001), calculated using 2-sample *t* test. **C)** Representative Col-0 root phenotypes 8 d after stratification, grown on the indicated concentrations of standard MS media (M0222, Duchefa) and isoxaben or mock.

As the isoxaben-induced RSSR is nutrient-dependent, we wondered whether this also held true for isoxaben-induced transcriptional responses. To test this, RNA-Seq was performed with roots harvested 6 d after stratification from seedlings grown on modified 1× MS media containing 6 mM or 60 mM KNO_3_ with 2.5 nM isoxaben or mock, referred to as long-term treatment. In parallel, another set of seedlings was grown on modified 1× MS media containing 6 mM or 60 mM KNO_3_ for 5 d and shifted to media containing the same concentration of KNO_3_ combined with either 2.5 nM isoxaben or mock, followed by root harvest at Day 6, referred to as short-term treatment. Under the short-term treatment, 145 and 1,605 differentially expressed genes (DEGs) were responsive to isoxaben at 6 mM and 60 mM KNO_3_, respectively, whereas the long-term treatment identified 827 and 4,522 DEGs as being isoxaben-responsive at 6 mM and 60 mM KNO_3_, respectively (Fig. 2A). Despite maintaining the same isoxaben concentration for both KNO_3_ media concentrations, the drastic difference in the number of isoxaben-responsive DEGs between 6 mM and 60 mM KNO_3_ provides further support that the isoxaben response is strongly influenced by media nutrient composition.

**Figure 2.**
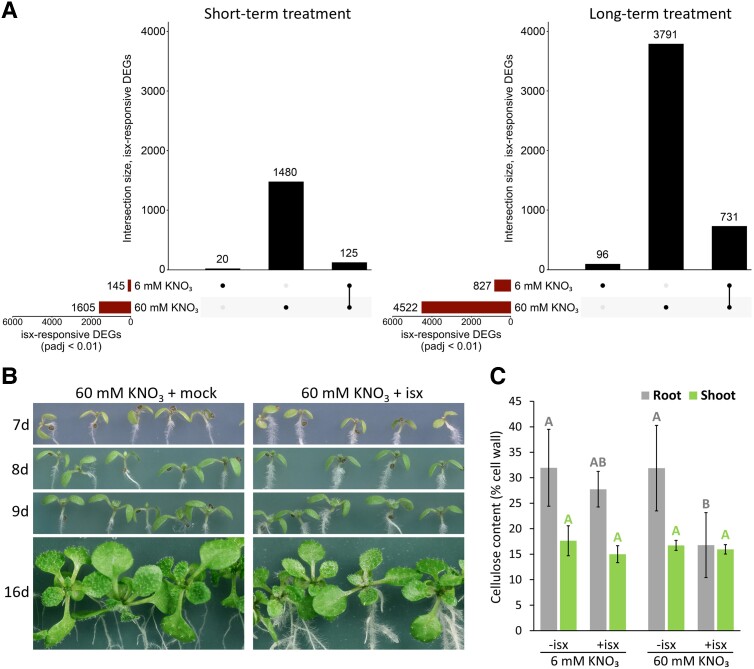
The phenotypic response to isoxaben is tissue-specific, and the transcriptional response is nutrient-dependent. **A)** RNA-Seq analysis of Col-0 roots from seedlings grown vertically on modified 1× MS media and harvested at 6 d after stratification. For short-term treatment, seedlings were grown on media containing 6 mM or 60 mM KNO_3_, and 5 d after stratification, they were shifted to media containing the same concentration of KNO_3_ with either 2.5 nM isoxaben or mock. Roots were harvested 24 h later on Day 6. For long-term treatment, seedlings were grown on media containing 6 mM or 60 mM KNO_3_ with 2.5 nM isoxaben or mock, and roots were harvested at 6 d after stratification. RNA was extracted using a commercial kit, libraries were prepared using poly(A) enrichment, and strand-specific 150 bp paired-end sequencing was carried out. Values indicate the number of DEGs showing a specific response to 2.5 nM isoxaben (*padj* < 0.01, *n* = 3). See Supplemental Methods for a detailed overview of the experimental setup and data analysis. **B)** Representative Col-0 shoot phenotypes from seedlings grown on modified 1× MS media containing 60 mM KNO_3_ and 2.5 nM isoxaben (isx) or mock (ethanol). Seedlings were imaged at the indicated number of days after stratification (d) from 4 independent growth assays. **C)** Crystalline cellulose quantification from Col-0 seedlings grown on modified 1× MS containing 6 mM or 60 mM KNO_3_ and 2.5 nM isx or mock (-isx). Tissue was harvested 10 d after stratification and cellulose quantified using a variation of the standard Updegraff method (see Supplemental Methods). Different letters indicate statistically different values within each tissue type (*P* < 0.05), calculated using 1-way ANOVA followed by Tukey's post hoc test. Error bars represent means ± Sd (*n* = 5).

While observing vertically grown seedlings on modified 1× MS media containing 60 mM KNO_3_ and up to 2.5 nM isoxaben, we noted another phenotypic peculiarity. Although isoxaben induced a clear RSSR, seedling shoot size and developmental staging were not obviously impaired even up to 16 d after stratification (Fig. 2B). When seedlings were germinated on higher concentrations of isoxaben, up to 7 nM for 7 d of growth, the RSSR became more pronounced, but shoot developmental processes appeared to be largely unaffected, including cotyledon emergence and expansion, the initiation of first true leaves, and trichome development (Supplemental Figs. S1 and S2). The same primary cell wall CESAs that are presumably inhibited in roots are also active in shoots, such as CESA1, which is required for proper trichome development. As CESAs are essential for cell and tissue development, their inhibition should have strong impacts on both root and shoot growth and development. Isoxaben is transported from roots to shoots in dicots and monocots (Salihu et al. 1998; Brabham et al. 2018), and it is not significantly metabolized (Heim et al. 1991; Schneegurt et al. 1994; Salihu et al. 1998; Brabham et al. 2018). Based on this knowledge, and combined with the fact that vertically grown shoots are in nearly constant contact with isoxaben-containing media, it is a near certainty that shoot cells are exposed to isoxaben. These results suggest that isoxaben may have tissue-specific activity.

To test whether isoxaben differentially affects shoots and roots, we grew seedlings on modified 1× MS media containing 6 mM or 60 mM KNO_3_ with 2.5 nM isoxaben or mock. Ten days after stratification, roots and shoots were separated, and crystalline cellulose was quantified. Shoot cellulose content was stable across all conditions, whereas cellulose content was only significantly decreased in roots grown on 60 mM KNO_3_ with isoxaben (Fig. 2C). These findings support the observation that isoxaben has a strong effect on roots with little effect on shoots.

Under the conditions tested in our assays, we demonstrated that the isoxaben response is both nutrient-dependent and tissue-specific in Arabidopsis. This raises a particular concern for research in plant mechanobiology and cell wall integrity fields, where isoxaben is used as a central tool to induce cell wall stress. Sweeping conclusions from studies in these fields may need to be taken with caution. For example, a model for cell wall damage response was developed from isoxaben-based experiments using root-based readouts from seedlings grown under multiple media compositions and incorporating whole seedling-derived gene expression data (Denness et al. 2011). Data used to generate such a model could be heavily skewed considering our findings of the tissue-specific and nutrient-dependent nature of the isoxaben response.

Taken together, we stress caution in relying on CBIs as main tools in cell wall studies until we gain a deeper understanding of the CBI mechanism of action. Furthermore, we note the importance of establishing standardized growth conditions to facilitate reproducible, comparable results between researchers. As nutrient availability can significantly influence isoxaben responses, attention must be paid to growth setups, as even the gelling agent used to prepare media can greatly alter the final media nutrient composition (Gruber et al. 2013). We note that 2.5 nM isoxaben can induce a strong RSSR and significant changes to the transcriptome, yet there is a staggering variation in the concentrations of isoxaben currently used across laboratory studies. From forward genetic screens using long-term growth on 20 nM isoxaben (Shim et al. 2018), hour-long treatments using 600 nM isoxaben (Engelsdorf et al. 2018), to treatment with 20 to 40 *µ*M isoxaben for 20 h or longer (Heisler et al. 2010; Sampathkumar et al. 2019), there is no established standard for the concentration and duration of CBI treatments. As a strong phenotypic response can be observed from even 1.5 nM isoxaben, the use of drastically high concentrations of CBIs (or any inhibitor) is almost certain to enhance off-target effects, masking true CBI-dependent responses.

We hope this article establishes the need for reconsidering the use of CBIs in future studies and for the potential reinterpretation of CBI-based studies as we learn more about the CBI mechanism of action.

##  

### Accession number

The RNA-Seq data set is available in the Gene Expression Omnibus (GEO) database under accession number GSE228764.

## Supplemental data

The following materials are available in the online version of this article.

**Supplemental Figure S1.** Representative images of Col-0 seedlings germinated on modified 1× MS media containing 60 mM KNO_3_ and varying concentrations of isoxaben, imaged 7 d after stratification.

**Supplemental Figure S2.** Representative images of Col-0 seedlings from Supplemental Fig. S1 captured under 100× magnification.

**Supplemental Table S1.** RNA-Seq gene expression data using root tissue grown during short-term and long-term 2.5 nM isoxaben treatments on modified 1× MS media containing 6 mM or 60 mM KNO_3_.

**Supplemental Methods.** Growth conditions, media composition, imaging and root measurement, crystalline cellulose quantification, and RNA-Seq experimental setup and data analysis.

## Supplementary Material

kiad538_Supplementary_Data

## Dive Curated Terms

The following phenotypic, genotypic, and functional terms are of significance to the work described in this paper:

GSE228764 Gramene: https://www.ncbi.nlm.nih.gov/geo/query/acc.cgi?acc=GSE228764
GSE228764 Araport: https://www.ncbi.nlm.nih.gov/geo/query/acc.cgi?acc=GSE228764

isoxaben CHEBI: CHEBI:63956

CESA Gramene: Cellulose synthase
CESA Araport: Cellulose synthase

GEO Gramene: https://www.ncbi.nlm.nih.gov/geo/
GEO Araport: https://www.ncbi.nlm.nih.gov/geo/
