# Supplementary material for: Cellulose biosynthesis inhibitor isoxaben causes nutrient-dependent and tissue-specific Arabidopsis phenotypes
Source: Plant Physiol. 2023 Oct 12;194(2):612–7. doi: 10.1093/plphys/kiad538 (PMC10828196; doi:10.1093/plphys/kiad538)
Supplement: kiad538_Supplementary_Data [file kiad538_supplementary_data.zip › SuppMethods.pdf]

Cellulose biosynthesis inhibitor isoxaben causes nutrient-dependent and tissue-specific Arabidopsis phenotypes

Michael Ogden, Sarah Whitcomb, Ghazanfar Abbas Khan, Ute Roessner, Rainer Hoefgen, Staffan Persson

Supplemental Methods

Growth conditions

Arabidopsis thaliana Col-0 was used for all experiments. Chlorine gas was used to sterilize seeds. Poly Klima growth cabinets were used for all growth experiments, with uniform LED lighting set to an intensity of 100 µmol/m^2/s, 16 hours light (21°C) and 8 hours dark (19°C). All seedlings were grown vertically in 12 cm petri plates that were positioned in custom-built acrylic racks to maintain consistent spacing between plates and a vertical orientation at a 5° angle.

Media composition

Unless otherwise stated, all growth assays were carried out using 1x Murashige & Skoog Modified Basal Salt Mixture (0.61 g/L) (M407, Phytotech Labs), which lacks nitrogen, phosphorus, potassium, and vitamins. All media was prepared using MilliQ water. Phosphorus was supplied as KH_2PO_4 to a final concentration of 1.35 mM, and 1 % (w/v) sucrose was included. Specific nutrients were added to desired final concentrations. The media was buffered using MES hydrate (0.5 g/L) and adjusted to pH 5.7 with 1 M KOH. Agar (A1296, Sigma) was added at 8 g/L. Following autoclaving, media was moved to a 60°C oven to stabilize the temperature across all bottles. Immediately prior to pouring media, MS vitamins were added from a sterile 1000x concentrated solution (1 mL/L) (M3900-50mL, Sigma), and isoxaben (36138-100MG, Sigma) was added from a 20 µM stock in 100 % anhydrous ethanol. Mock treatment consisted of an equivalent volume of 100 % anhydrous ethanol. Each 12 cm square petri plate (688102, Greiner Bio-One) was filled with 100 mL of media. After sowing seeds, plates were sealed with micropore tape and stratified in the dark at 4°C for three days.

Standard MS with vitamins (M0222, Duchefa) was prepared using MilliQ water, 1 % (w/v) sucrose, buffered using MES hydrate (0.5 g/L), and adjusted to pH 5.7 with 1 M KOH. Agar (A1296, Sigma) was added at 8 g/L. Following autoclaving, media was held in an oven at 60°C, and isoxaben or mock treatment was added immediately prior to pouring.

Imaging and root measurement

Vertically grown seedlings on agar plates were imaged using an Epson V850 Pro scanner, and primary root lengths were measured using WinRHIZO (Regent Instruments). A Keyence VHX-6000/7000 digital microscope was used to capture high resolution images of seedlings and root tips. Minitab 20 was used for statistical analysis.

Crystalline cellulose quantification

One row of Col-0 seedlings was grown per square plate with modified 1x MS media containing 6 mM or 60 mM KNO_3 with 2.5 nM isoxaben or mock. Ten days after stratification, vertically grown seedlings were harvested by separating roots and shoots with a razor blade. Per plate, roots and shoots were separately pooled and moved to 2 mL microcentrifuge tubes containing 1.5 mL of 70% ethanol. Pooled tissue from each plate was considered a biological replicate, with a minimum of five replicates

analyzed per condition. Crystalline cellulose was quantified using a modern variation of the standard Updegraff method (Updegraff, 1969), as outlined by Kumar & Turner (2015). The protocol was optimized for handling fragile seedling roots and shoots, which are prone to sample loss during various washing and aspiration steps. This was accomplished by replacing glass tubes with 15 mL conical polypropylene centrifuge tubes, which allowed sample centrifugation at 2,000 x g for 10 min between steps to pellet the samples. Minitab 20 was used for statistical analysis.

RNA-Seq experimental setup and data analysis

Roots were used for RNA-Seq analysis. Seedlings were grown vertically on 12 cm square petri plates containing modified 1x MS media, as described in the Growth Conditions and Media Composition sections.

To investigate the long-term effect of 2.5 nM isoxaben on the transcriptome, the growth conditions included: (**1**) 6 mM KNO_3 with mock, (**2**) 6 mM KNO_3 with isoxaben, (**3**) 60 mM KNO_3 with mock, and (**4**) 60 mM KNO_3 with isoxaben. Four more conditions were included that were designed to investigate the short-term impact of 2.5 nM isoxaben. Seedlings were grown on 6 mM or 60 mM KNO_3 (with mock control) and five days after stratification they were transferred at mid-day to media containing the same concentration of KNO_3 with either mock or isoxaben for 24 hours, and harvested along with all other samples at midday on day six. Therefore, the short-term treatment conditions included: (**5**) 6 mM KNO_3, transfer to mock, (**6**) 6 mM KNO_3, transfer to isoxaben, (**7**) 60 mM KNO_3, transfer to mock, and (**8**) 60 mM KNO_3, transfer to isoxaben.

The experimental setup was as follows: Col-0 seed were sterilized with chlorine gas, and one row of seeds (approximately 30-40 seeds) was sown per petri plate. For conditions (**1**) – (**4**), seeds were sown directly on agar, whereas for conditions (**5**) – (**8**) seeds were sown on an autoclaved polyester mesh (30 µm, #2-4072, NeoLab) placed on the agar. After three days of stratification, plates were moved to a growth cabinet and grown vertically. For conditions (**5**) – (**8**), five days after stratification, at midday, sterile forceps were used to quickly transfer the mesh (containing seedlings) to mock or treatment plates, which were sealed with micropore tape and returned to the growth cabinet. After 24 hours, at midday six days after stratification, roots from each plate across all conditions were quickly separated using a razor blade, moved to a 2 mL Safe-Lock microcentrifuge tube containing one 5 mm zirconia ball, and rapidly frozen in liquid nitrogen. Each plate was treated as a biological replicate. Three replicates were sequenced per condition.

A Retsch Mill was used to grind frozen root tissue and an RNeasy Plant Mini Kit (Qiagen) was used to extract RNA from three samples per condition. RNA concentration and quality were assessed using a BioAnalyzer 2100 (Agilent). Library prep (poly A enrichment) and strand-specific 150 bp paired-end sequencing were carried out by BGI (Hong-Kong) using the DNBSEQ platform.

Raw sequencing reads were cleaned by BGI using their SOAPnuke software, and approximately 20 million cleaned reads per sample were delivered. The quality of these cleaned reads was checked using FastQC (https://www.bioinformatics.babraham.ac.uk/projects/fastqc/). Cleaned reads were mapped to the TAIR10 Arabidopsis thaliana genome, and TAIR10 release 51 genome annotated with STAR v2.7.9a (https://github.com/alexdobin/STAR). The quality of the mapping was assessed with MultiQC v1.10.1 (https://multiqc.info). To account for differences in sequencing depth and RNA composition between samples, the reads per gene were normalized by the median of ratios method in the DESeq2 v1.32.0 R package (https://bioconductor.org/packages/release/bioc/html/DESeq2.html). Differential expression analysis, including automated shrinkage of exaggerated log2FoldChanges using

the ashr method (Stephens, 2017), was also performed with DEseq2 v1.32.0. The R package UpSetR (https://cran.r-project.org/web/packages/UpSetR/index.html) was used to visualize DEG set intersections.

Supplemental References

Kumar M, Turner S (2015) Protocol: a medium-throughput method for determination of cellulose content from single stem pieces of Arabidopsis thaliana. Plant Methods **11**: 46

Stephens M (2017) False discovery rates: a new deal. Biostatistics **18**: 275-294

Updegraff DM (1969) Semimicro determination of cellulose in biological materials. Anal Biochem **32**: 420-424
