# Supplementary figures and images for: Cellulose biosynthesis inhibitor isoxaben causes nutrient-dependent and tissue-specific Arabidopsis phenotypes
Source: Plant Physiol. 2023 Oct 12;194(2):612–7. doi: 10.1093/plphys/kiad538 (PMC10828196; doi:10.1093/plphys/kiad538)

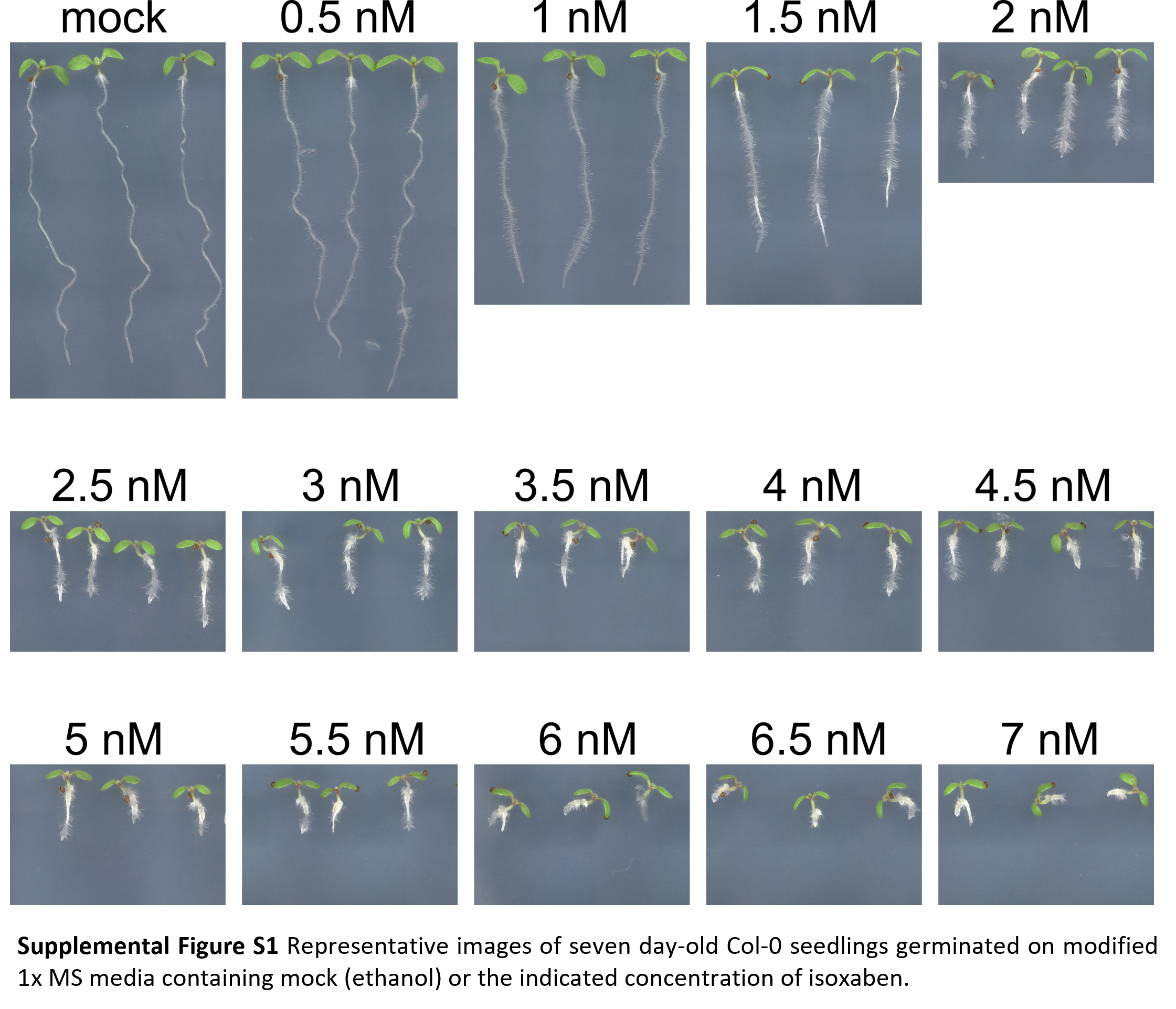

Supplement: kiad538_Supplementary_Data [file kiad538_supplementary_data.zip › Fig S1.tif]

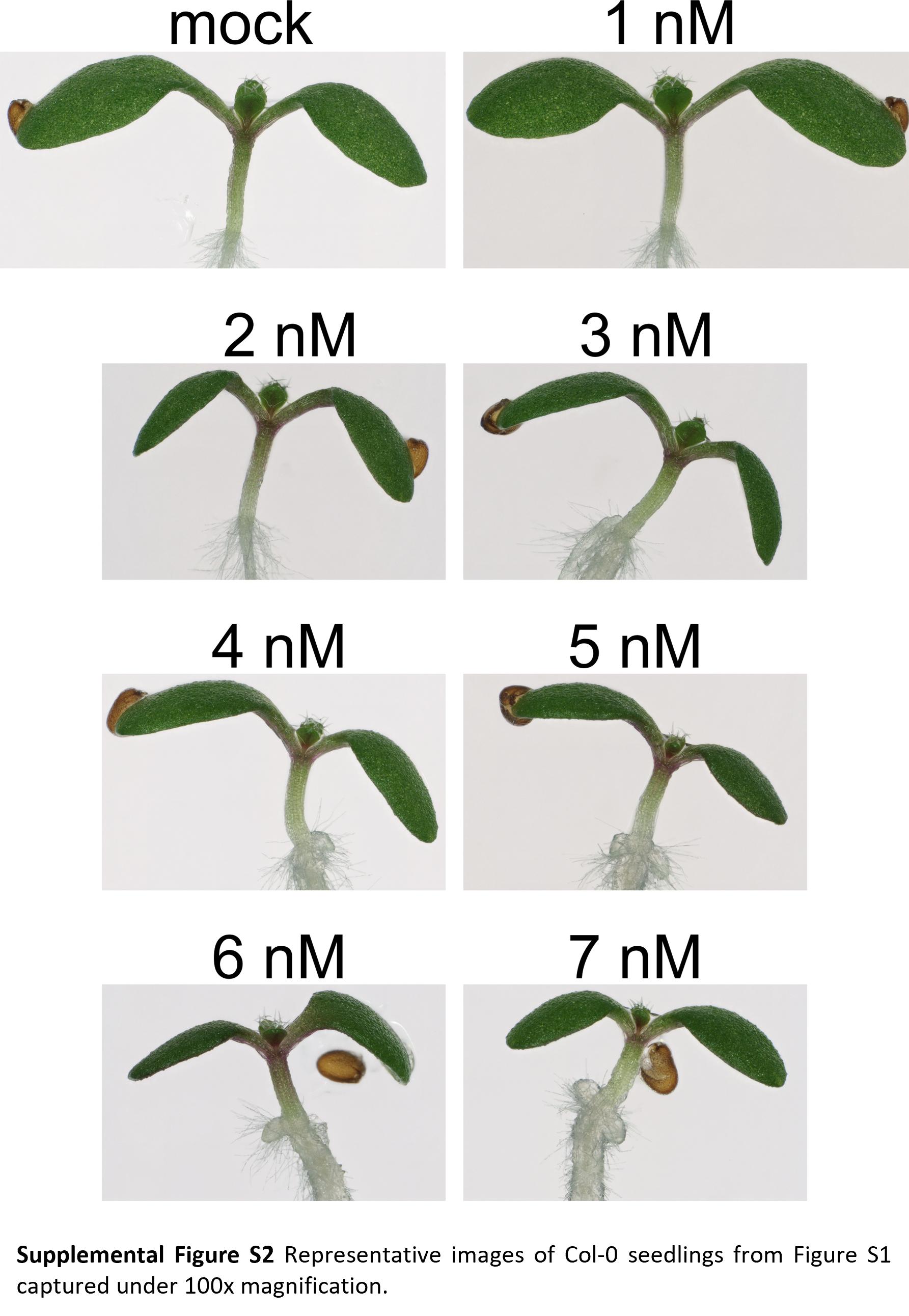

Supplement: kiad538_Supplementary_Data [file kiad538_supplementary_data.zip › Fig S2.tif]
